# A pilot study on coronary microvascular dysfunction in obstructive hypertrophic cardiomyopathy: impact of percutaneous transluminal septal myocardial ablation

**DOI:** 10.1007/s12928-025-01154-1

**Published:** 2025-06-27

**Authors:** Taikan Terauchi, Daigo Hiraya, Kyohei Usami, Takumi Yaguchi, Hiroaki Watabe, Tomoya Hoshi, Tomoko Ishizu

**Affiliations:** Department of Cardiology, Institute of Medicine, University of Tsukuba, 1-1-1 Tennodai, Tsukuba, 3058575 Japan

**Keywords:** Hypertrophic cardiomyopathy, Coronary microvascular dysfunction, Septal ablation, Coronary flow reserve, Microvascular resistance

## Abstract

Coronary microvascular dysfunction (CMD) is well-characterized in the context of coronary artery disease, but its relationship to obstructive hypertrophic cardiomyopathy (oHCM) is poorly understood. In addition, the impact of percutaneous transluminal septal myocardial ablation (PTSMA) on CMD has not been fully evaluated. Between October 2023 and May 2024, PTSMA was performed on 10 patients with oHCM. A pressure guidewire in the left anterior descending artery (LAD) was used to invasively assess CMD before and after the procedure. Measurements were recorded for resting full-cycle ratio (RFR), fractional flow reserve (FFR), coronary flow reserve (CFR), and index of microcirculatory resistance (IMR). The 10 patients had a median age of 66 [57–75] years, with a resting left ventricular pressure gradient of 44 [17–84] mmHg, measured via catheterization. Prior to PTSMA, the RFR measured in the LAD was 0.93 [0.91–0.96], and the FFR was 0.95 [0.92–0.95], which were both within normal limits. However, the CFR was reduced to 1.8 [1.6–2.1], and the IMR was elevated to 31 [25–39], which indicated CMD. Post-procedure, the left ventricular pressure gradient decreased to 5 [2–8] mmHg, CFR improved to 2.5 [2.2–3.6], and IMR decreased to 22 [17–26], indicating improvement in CMD. In patients with oHCM, myocardial hypertrophy contributes to left ventricular outflow tract obstruction and CMD. This study demonstrated that PTSMA as a septal reduction therapy improved the left ventricular pressure gradient and CMD.

**Graphical abstract:**

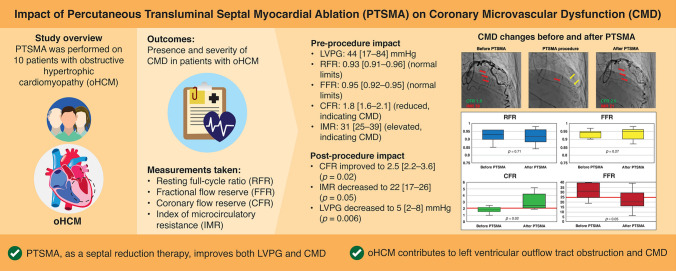

## Introduction

Coronary microvascular dysfunction (CMD) is defined as structural and functional abnormalities in microvessels smaller than 500 μm. This condition is associated with various cardiovascular diseases and is increasingly recognized as a cause of angina [1–4]. Many patients with angina undergo coronary computed tomography angiography or coronary angiography to evaluate epicardial vessels; however, CMD cannot be assessed using these modalities [5, 6]. Nevertheless, the presence of CMD has been linked to significantly increased mortality and coronary events [7, 8].

Traditional, non-invasive methods to assess coronary microvascular function have included coronary flow reserve (CFR) in the left anterior descending artery (LAD) using Doppler flow echocardiography, myocardial blood flow, and myocardial flow reserve (MFR) evaluation with cardiac magnetic resonance imaging (cMRI), or positron emission tomography (PET). More recently, invasive diagnostic techniques have been developed that can directly assess CMD via a coronary guidewire. Patients can then be classified based on endotype, which allows for the development of more targeted therapeutic interventions to reduce cardiovascular events and improve quality of life [9–12].

Hypertrophic cardiomyopathy (HCM) has long been associated with CMD because of increased myocardial oxygen demand, elevated myocardial wall tension, impaired capillary dilation, and increased resting coronary flow [13–15]. Usually detected by cMRI or PET, CMD is a hallmark of HCM and an independent prognostic factor [16, 17]. One form of HCM, obstructive hypertrophic cardiomyopathy (oHCM), is characterized by particularly severe CMD [18]. Surgical myectomy or percutaneous transluminal septal myocardial ablation (PTSMA) are well-established interventions for symptomatic patients with oHCM who have a substantial drug-resistant pressure gradient (PG) [19, 20]. However, research on invasive investigation of CMD in patients with oHCM remains limited, and the impact of PTSMA on CMD is poorly understood. This study aimed to use invasive physiological measurements to assess the presence and severity of CMD in patients with oHCM. In addition, the study evaluated the impact of PTSMA on CMD by analyzing pre- and post-procedural changes in CFR and the index of microcirculatory resistance (IMR).

## Methods

### Data source and study population

Between October 2023 and May 2024, PTSMA was performed on 10 patients with oHCM at the University of Tsukuba Hospital. The diagnosis followed the Japanese Circulation Society guidelines [21], and patients were evaluated for PTSMA eligibility based on standard criteria. Echocardiography or cMRI was used to exclude patients with cardiomyopathies and to confirm that all patients had left ventricular (LV) hypertrophy (≥ 15 mm) that was not attributable to pressure overload conditions such as hypertension or aortic valve stenosis. Endomyocardial biopsy or cMRI was also used to exclude patients with secondary cardiomyopathies.

All patients were in New York Heart Association (NYHA) functional class II or III and had a left ventricular outflow tract (LVOT) PG ≥ 50 mmHg, measured via echocardiography or catheterization.

The study was conducted in accordance with the Declaration of Helsinki and was approved by the Tsukuba University Ethics Committee (approval number: R06-167).

### Invasive assessment of coronary microvascular dysfunction

A standardized invasive protocol using a 7-French guiding catheter inserted via the femoral artery was used to assess CMD before and after PTSMA. A 4-French MTAKA (Nipro Corp., Osaka, Japan) catheter was introduced through the right radial artery to measure LVOT-PG between the left ventricle and the aorta.

The CoroFlow Cardiovascular System (Coroventis Research AB, Uppsala, Uppsala County, Sweden) was used to assess CMD. A PressureWire™ X (St. Jude Medical, Atrial Fibrillation Division, Inc., St. Paul, Minnesota, United States) was inserted into the LAD and equalized at the left main coronary artery before being advanced into the distal LAD. The following coronary physiologic indices were measured: resting full-cycle ratio (RFR), CFR, IMR, and fractional flow reserve (FFR).

**Fig. 1:**
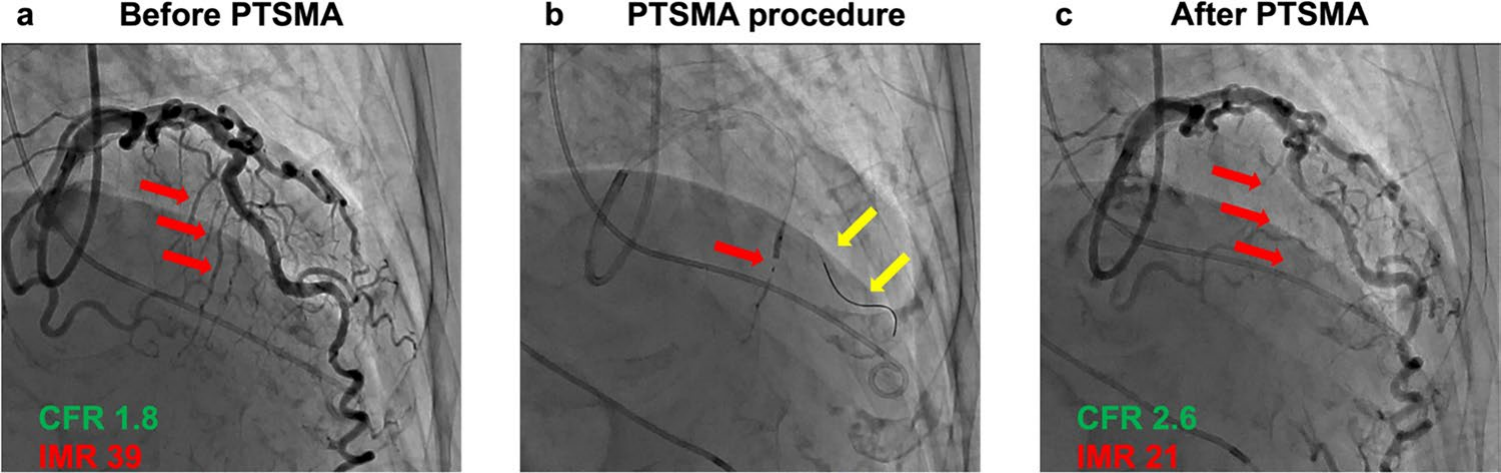
Representative case of coronary microvascular dysfunction changes before and after percutaneous transluminal septal myocardial ablation. **a** Patient with oHCM exhibiting CMD. Red arrow: Target septal branch. b: PTSMA performed by injecting ethanol into the target septal branch while keeping the pressure wire inserted in the LAD. Red arrow: Ethanol injection into the target septal branch. Yellow arrow: Pressure wire. c: CMD improved after PTSMA. Red arrow: Disappearance of the target septal branch. *CMD* coronary microvascular dysfunction, *PTSMA* percutaneous transluminal septal myocardial ablation, *oHCM* obstructive hypertrophic cardiomyopathy, *CFR* coronary flow reserve, *IMR* index of microcirculatory resistance, *LAD* left anterior descending artery

The CFR and IMR were assessed using three 5 cc injections of saline at room temperature, followed by 2 mg of nicorandil infusion into the left coronary artery to induce maximal hyperemia. Three additional 5 cc saline injections were administered to determine hyperemic transit time. Three cardiologists adjudicated all transit time measurements; if any value was deemed an outlier, the measurement was repeated. The CoroFlow system was used to calculate CFR and IMR based on measured transit times. Values of ≥ 2.0 for CFR and ≤ 25 for IMR were considered normal [22]. The FFR was also assessed, and drift correction was performed at the left main coronary artery to ensure accuracy. Once stability was confirmed, the PressureWire™ was repositioned in the distal LAD, where it remained throughout PTSMA. After the procedure, the RFR, FFR, CFR, and IMR were reassessed using the same protocol to evaluate post-procedural changes in coronary microvascular function (Fig. 1).

### Percutaneous transluminal septal myocardial ablation procedure

The PTSMA procedure was performed following Japanese Circulation Society guidelines. To prevent atrioventricular (AV) block complications, a temporary pacemaker was placed in the right ventricle via a 5-French sheath inserted into the right internal jugular vein.

The target septal branch was identified using pre-procedural echocardiography and coronary angiography. A guidewire was inserted into the target septal branch, followed by an over-the-wire balloon or microcatheter. A small contrast injection ensured that there was no leakage into the LAD. Transthoracic contrast echocardiography was performed to confirm the target area.

Following analgesic administration, ethanol was injected into the target septal branch; the volume was adjusted based on the perfusion area. Immediately after PTSMA, the LVOT-aorta PG was reassessed. One week post-procedure, echocardiography was repeated to measure the LVOT-PG and LV septal wall thickness. BNP levels were also measured 1 week after the procedure.

### Statistical analysis

Because of the small sample size, continuous variables were analyzed using nonparametric tests and are presented as median [interquartile range]. Categorical variables are expressed as frequency (percentage). Comparisons of continuous variables were performed using the Wilcoxon signed-rank test. A p-value of < 0.05 was considered statistically significant.

Statistical analyses were performed using EZR software version 1.68 (Saitama Medical Center, Jichi Medical University, Saitama, Japan), a graphical interface for R (The R Foundation for Statistical Computing, Vienna, Austria). This software is a modified version of R Commander, incorporating statistical functions frequently used in biostatistics.

## Results

### Baseline characteristics

The study included 10 patients with a median age of 66 [57–75] years, of whom three (30%) were male. The patients had NYHA functional classifications of II or III, and the median brain natriuretic peptide (BNP) level was 156 [65–244] pg/mL. Echocardiography showed a median interventricular septum thickness (IVST) of 15.0 [13.7–16.9] mm, which confirmed LV septal hypertrophy in all patients. Seven patients (70%) had isolated LVOT obstruction, whereas three (30%) had an obstruction from the mid-ventricular area to the LVOT. The median resting PG was 39 [17–79] mmHg, and three patients (30%) exhibited mitral regurgitation (MR) due to systolic anterior motion (SAM) of the mitral valve.

All patients were on β-blockers; some were also receiving cibenzoline or calcium channel blockers. Preoperative cMRI was performed on eight patients; late gadolinium enhancement (LGE) was revealed in five (63%), and apical aneurysm was found in two (25%). The median LV mass index was 63.1 [55.3–73.4] g/m^2^ **(**Table 1**)**.

**Table 1:** Baseline characteristics of patients with obstructive hypertrophic cardiomyopathy

Baseline characteristics variable	Value (n = 10)
Age, years	66 [57–75]
Male, n (%)	3 (30)
BSA, m^2^	1.56 [1.43–1.72]
Systolic BP, mmHg	125 [118–132]
Diastolic BP, mmHg	72 [68–76]
HR, bpm	74 [66–78]
Hypertension, n (%)	7 (70)
Diabetes, n (%)	1 (10)
Dyslipidemia, n (%)	4 (40)
Chronic kidney disease, n (%)	2 (20)
Family history, n (%)	0 (0)
Atrial fibrillation, n (%)	3 (30)
LVOT obstruction, n (%)	7 (70)
MV obstruction, n (%)	3 (30)
NYHA class IIs	0 (0)
NYHA class IIm	4 (40)
NYHA class III	6 (60)
HCM Risk-SCD score, (%)	1.91 [1.49–2.31]
Hb, g/dL	13.2 [11.9–14.3]
BNP, pg/mL	156 [65–244] ^a^
**Echocardiography findings (n = 10)**	**(n = 10)**
LVEF, %	67 [64–72]
Maximum wall thickness, mm	16.8 [15.7–17.0]
IVST, mm	15.0 [13.7–16.9]
LAVI, mL/m^2^	51 [45–57]
E/A ratio	0.8 [0.7–1.0] ^b^
E/e’ ratio	12.7 [9.3–16.2] _c_
LVOT gradient at rest, mmHg	39 [17–79] ^d^
SAM-MR, n (%)	3 (30)
**Medication use (n = 10)**	**(n = 10)**
β blocker, n (%)	10 (100)
Cibenzoline, n (%)	6 (60)
Calcium channel blockers, n (%)	6 (60)
**Cardiac MRI findings (n = 8)**	**(n = 8)**
LGE, n (%)	5 (63)
Aneurysm, n (%)	2 (25)
LVMI, g/m^2^	63.1 [55.3–73.4]
LVEDV, mL	146 [134–189]
LVESV, mL	65 [47–86]
SV, mL	90 [78–101]
CO, L/min	5.4 [4.2–6.7]
CI, L/min/m^2^	3.5 [3.0–4.1]
HR, bpm	59 [56–61]
LVEF, %	60 [56–66]

Values are expressed as median [interquartile range] or n (%)

^a^BNP values are presented as median [interquartile range] because of non-normal distribution

^b^E/A ratio is used as an indicator of diastolic function

^c^E/e’ ratio represents left ventricular filling pressures

^d^LVOT gradient measured via Doppler echocardiography

*BSA* body surface area, *BP* blood pressure, *HR* heart rate, *LVOT* left ventricular outflow tract, *MV* midventricular, *NYHA* New York Heart Association, *SCD* sudden cardiac death, *Hb* hemoglobin, *BNP* brain natriuretic peptide, *LVEF* left ventricle ejection fraction, *IVST* interventricular septum thickness, *LAVI* left atrial volume index, *E*/*A* early to diastolic mitral inflow velocity ratio, E/e’ ratio of early mitral inflow velocity to mitral annular velocity, *SAM* systolic anterior movement, *LGE* late gadolinium-enhancement, *LVMI* left ventricular mass index, *LVEDV* left ventricular end-diastolic volume, *LVESV* left ventricular end-systolic volume, *SV* stroke volume, *CO* cardiac output, *CI* cardiac index

### Physiological assessment and procedure summary

Right heart catheterization confirmed normal hemodynamic parameters in all patients, with no evidence of elevated filling pressures. The median pre-PTSMA RFR in the LAD was 0.93 [0.91–0.96], and the median FFR was 0.95 [0.92–0.95], both of which were within normal ranges. However, the median CFR was reduced to 1.8 [1.6–2.1], and the median IMR was elevated at 31 [25–39], indicating CMD (Fig. 1).

The PTSMA procedure was successfully performed in all patients. The median number of target septal branches was 3 [2, 3], and the maximum balloon size was 2.0 [2.0–2.0] mm. The median total ethanol volume was 4.2 [3.5–5.1] cc, and the post-procedure peak creatine kinase level reached 1634 [1532–2102] IU/L (Table 2).

**Table 2:** Percutaneous transluminal septal myocardial ablation procedure summary and right heart catheterization

PTSMA variable (n = 10)	Value
Number of target septal branches	3 [2, 3]
Maximum balloon size, mm	2.0 [2.0–2.0]
Total ethanol, mL	4.2 [3.5–5.1]
Peak Creatine Kinase, IU/L	1634 [1532–2102]
Peak Creatine Kinase-MB, IU/L	229 [201–332]
Right heart catheterization (n = 10)	
RA, mmHg	6.5 [4.5–7.8]
RV, mmHg	9.0 [7.3–10.8]
mPAP, mmHg	18.5 [17.3–22.3]
PCWP, mmHg	13.5 [12.3–15.8]
CO, L/min	4.6 [3.9–5.8]
CI, L/min/m^2^	3.1 [2.6–3.7]

Values are expressed as median [interquartile range]

*PTSMA* percutaneous transluminal septal myocardial ablation, *RA* right atrium, *RV* right ventricle, *mPAP* mean pulmonary arterial pressure, *PCWP* pulmonary artery wedge pressure, *CO* cardiac output, *CI* cardiac index

Regarding AV block, there was only one case in which transient complete AV block occurred during the PTSMA procedure; however, the patient returned to sinus rhythm before the end of the treatment. The median resting PG, measured by catheterization before and after PTSMA, improved from 44 [17–84] mmHg to 5 [2–8] mmHg (p = 0.006). The median PG during the Brockenbrough maneuver decreased from 126 [100–165] mmHg to 7 [4–26] mmHg (p = 0.002). One week post-PTSMA, the median echocardiographic PG decreased to 15 [10–21] mmHg (p = 0.009), and the median IVST decreased from 15.0 [13.7–16.9] mm to 12.4 [11.7–16.4] mm (Table 3).

**Table 3:** Comparison of clinical findings before and after percutaneous transluminal septal myocardial ablation

Hemodynamic and echocardiographic variables	Baseline	After PTSMA	p-value
Systolic BP, mmHg	125 [118–132]	125 [118–144]	0.96
Diastolic BP, mmHg	72 [68–76]	69 [60–71]	0.28
HR, bpm	74 [66–78]	65 [61–72]	0.22
**Electrocardiogram**			
Sinus rhythm, n (%)	10 (100)	10 (100)	–
Complete atrioventricular block	0 (0)	0 (0)	–
Atrial fibrillation	0 (0)	0 (0)	–
**Catheter measurements**			
LVOT pressure gradient at rest, mmHg	44 [17–84]	5 [2–8]	0.006**
LVOT pressure gradient (Brockenbrough maneuver), mmHg	126 [100–165]	7 [4–26]	0.002*
LVEDP, mmHg	18 [15–23]	20 [17–22]	0.61
**Echocardiography (After 1 week)**			
LVOT pressure gradient at rest, mmHg	39 [17–79]	15 [10–21]	0.009
IVST, mm	15.0 [13.7–16.9]	12.4 [11.7–16.4]	0.02
BNP, pg/mL	156 [65–244]	103 [63–193]	0.74

Values are expressed as median [interquartile range] or n (%)

p < 0.05, ** p < 0.01 (statistical significance defined as p < 0.05)

*LVOT* left ventricular outflow tract, *LVEDP* left ventricular end-diastolic pressure, *IVST* interventricular septum thickness, *BNP* brain natriuretic peptide, *PTSMA*: percutaneous transluminal septal myocardial ablation

### Coronary microcirculation immediately after percutaneous transluminal septal myocardial ablation

**Fig. 2:**
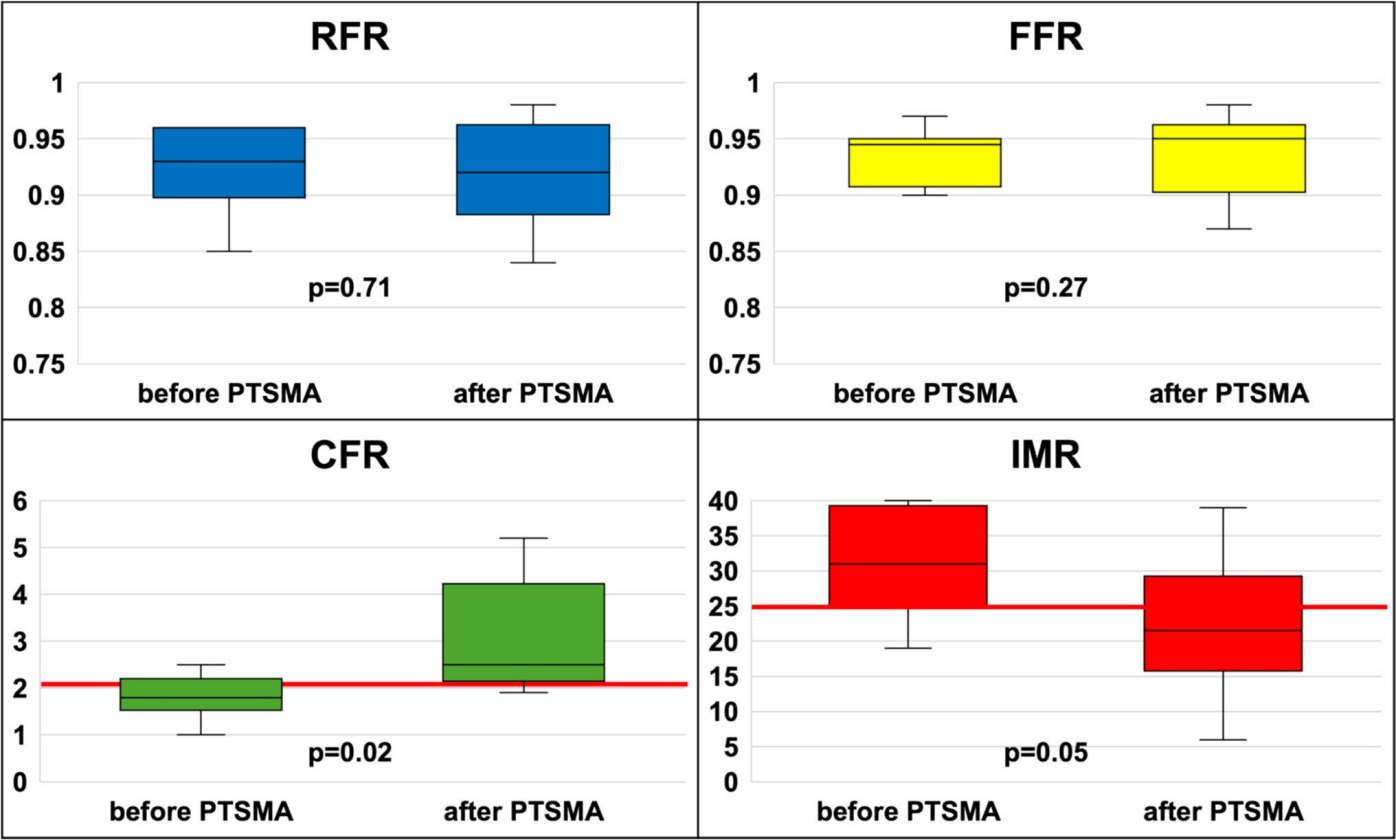
Evaluation of functional ischemia and coronary microvascular dysfunction before and after percutaneous transluminal septal myocardial ablation. *RFR* resting full-cycle ratio, *FFR* fractional flow reserve, *CFR* coronary flow reserve, *IMR* index of microcirculatory resistance

**Fig. 3:**
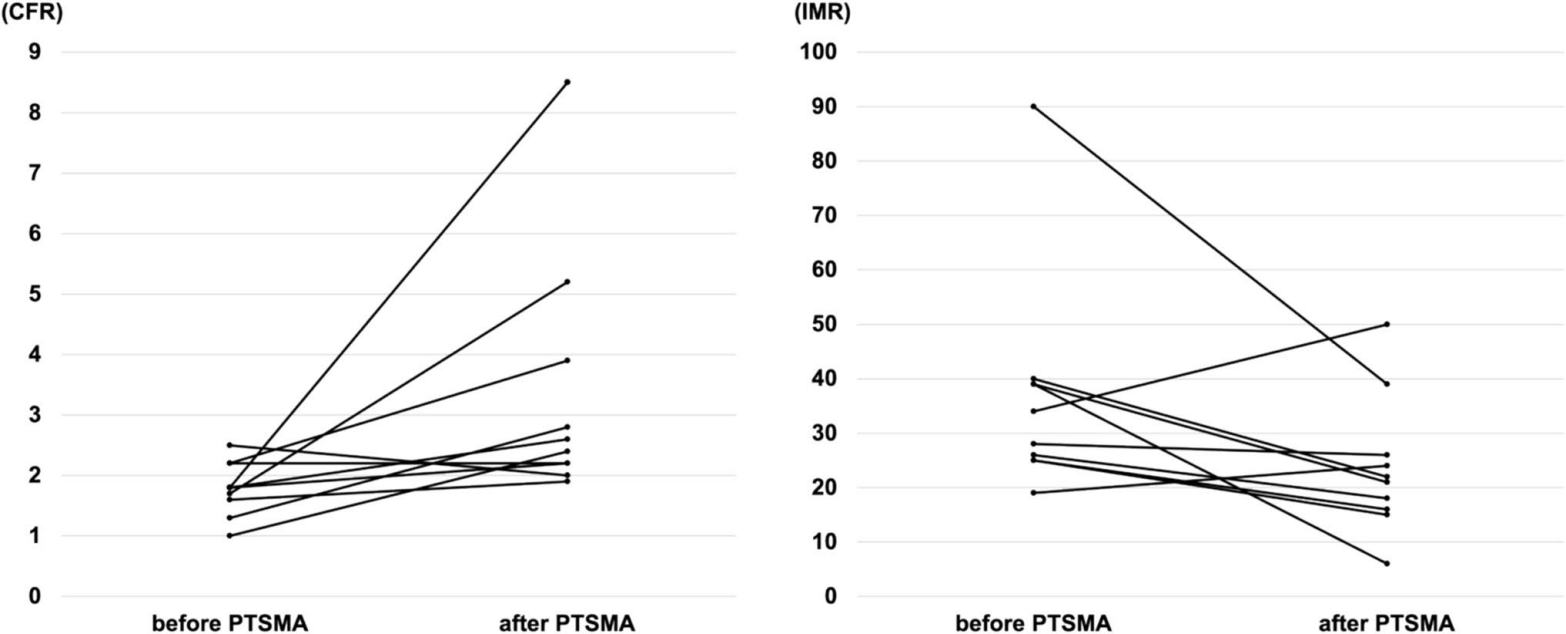
Evaluation of coronary microvascular dysfunction before and after percutaneous transluminal septal myocardial ablation. Individual changes in (**a**) coronary flow reserve and (**b**) index of microcirculatory resistance. *PTSMA* percutaneous transluminal septal myocardial ablation, *CFR* coronary flow reserve, *IMR* index of microcirculatory resistance

Values for RFR, FFR, CFR, and IMR were reassessed immediately after PTSMA using the same methodology as before treatment. The RFR values did not change (0.92 [0.89–0.96]; p = 0.71), nor did the median FFR change (0.95 [0.92–0.96]; p = 0.27). However, the median CFR increased to 2.5 [2.2–3.6] (p = 0.02), and the median IMR decreased to 22 [17–26] (p = 0.05), both of which indicated improved CMD (Figs. 2 and 3). SAM-MR was observed in three patients before treatment. In one case, where SAM-MR completely resolved after PTSMA, CMD showed particularly marked improvement, with CFR increasing from 1.7 to 5.2 and IMR decreasing from 26 to 18.

## Discussion

This study confirmed that CMD was prevalent in patients with oHCM and was associated with CMD. Data from the study also indicated that PTSMA led to immediate CMD improvement, as evidenced by an increase in CFR and a reduction in IMR. PTSMA is a therapeutic procedure that intentionally induces localized myocardial infarction by occluding septal branches. In the treated area, microcirculation is expected to be impaired because of the loss of capillary beds and reduced blood flow. Nevertheless, in this study, CFR improved, and IMR decreased after PTSMA, suggesting that the observed changes in these parameters reflect an overall improvement in coronary microvascular function that outweighs the localized effects at the treatment site. To the best of our knowledge, this is the first study to evaluate pre- and post-PTSMA CMD using a guidewire-based assessment in the LAD in patients with oHCM and substantial PG. Given the association between CMD and adverse cardiovascular outcomes, these findings highlight the potential therapeutic significance of PTSMA beyond its hemodynamic benefits [7, 8].

CMD in oHCM differs from classic microvascular angina, as pharmacological therapies such as β-blockers and calcium channel blockers are often insufficient to reverse microvascular dysfunction [23, 24]. In this study, CMD was present despite β-blocker therapy in all patients, suggesting a distinct pathophysiology. The presumed mechanisms involve increased myocardial oxygen demand resulting from hypertrophy, microvascular remodeling, myocardial fibrosis, and hypertrophy of vascular smooth muscle cells [25]. Hemodynamically, septal hypertrophy and LVOT obstruction contribute to extravascular compression of the coronary microvasculature, which further worsens CMD [26]. In this study, SAM-MR was observed in three patients before treatment. Notably, in the one patient in whom SAM-MR completely resolved after PTSMA, CMD showed particularly marked improvement. Although the small sample size limits generalizability, these findings suggest a potential association between SAM-MR and CMD. In this study, RFR and FFR showed no change before and after PTSMA. Unlike spontaneous myocardial infarction, PTSMA is a therapeutic procedure that does not involve stenosis or occlusion of the main trunk of the LAD and, therefore, has minimal impact on distal and aortic pressure measured in the LAD. In addition, the complete loss of perfusion to the target septal branches likely explains the lack of change in RFR and FFR before and after the procedure.

Conducting PTSMA resulted in a large improvement in CMD, as reflected by increased CFR and reduced IMR. This is supported by previous evidence that CFR is influenced by multiple hemodynamic confounding factors beyond the coronary arteries, such as cardiac workload, blood pressure, and heart rate [27–30].

In this study, PTSMA reduced the LVOT-PG, which suggested that the improvement in hemodynamics might have contributed to the increase in CFR. However, this study also demonstrated that PTSMA not only improved CFR but also led to an improvement in IMR, which was a specific indicator of coronary microvascular resistance. Therefore, there was likely a true improvement in coronary microvascular function independent of hemodynamic changes. Although guidewire-based CMD assessment is invasive, it has the advantage of evaluating IMR, which cannot be measured using cMRI or PET. The improvement in IMR following PTSMA is thought to be driven by the reduction in wall tension resulting from the alleviation of obstruction, which subsequently reduces extravascular compression of the microvasculature [26]. These changes are more likely attributable to immediate physiological effects—such as acute hemodynamic improvement and reduced extravascular compression caused by PTSMA—rather than chronic effects from structural reverse remodeling of the microvasculature. Therefore, the improvements in CMD indices observed immediately after PTSMA are considered to reflect the acute effects of the treatment. If AV block occurs during the PTSMA procedure, fluctuations in blood pressure may affect coronary microcirculation. In this study, one patient experienced transient complete AV block during the procedure; however, all patients were in sinus rhythm at the time of coronary microcirculation assessment. Therefore, we believe that changes in cardiac rhythm did not influence the results of coronary microcirculation measurements.

Despite improvements, some patients exhibited persistent CMD even with an IMR of 22 [17–26] post-PTSMA, which suggested that microvascular remodeling might have already advanced in some cases, potentially limiting further recovery. Because CMD has been shown to develop before adverse clinical outcomes in patients with oHCM, PTSMA may improve hemodynamics, potentially enhancing coronary microcirculation and contributing to better long-term prognosis [16]. However, residual CMD after PTSMA may represent a persistent risk factor, which points to the importance of post-treatment monitoring, including the consideration of more intensive pharmacological therapy.

Further studies are warranted to investigate the long-term effects of PTSMA on patients with CMD and the impact of intensive pharmacological treatment with novel agents such as mavacamten, not only on hemodynamics but also on microvascular function.

## Limitations

This study was a single-center retrospective analysis with a small sample size, which limits the generalizability of the findings. To ensure broader applicability, future studies with larger sample sizes and detailed stratification are necessary. Therefore, the findings of this study should be considered as those of a pilot study. This study did not include a comparator group, such as untreated patients or those who received alternative therapies; therefore, it is not possible to definitively determine whether the observed improvement in CMD was solely attributable to PTSMA or influenced by natural recovery or a placebo effect. In addition, whether the effects of PTSMA on CFR and IMR are sustained over time remains unclear; therefore, further investigation is warranted. Future studies should also explore the relationship between residual CMD after PTSMA and long-term clinical outcomes. Additionally, in this study, CMD was evaluated in the LAD using a pressure wire. However, this method may not sufficiently reflect regional microvascular function in the septal myocardium, which is the direct target of PTSMA. Further research is required to clarify the regional and global effects of PTSMA on coronary microvascular function, through invasive evaluation of CMD in septal branches and the use of advanced imaging modalities.

Selection bias might have also been present as a result of the handling of outliers in CMD measurements. In addition, the potential influence of sedation and analgesic medications on CMD remains uncertain. Further limitations included the lack of stratification based on heart failure severity, the absence of genetic mutation screening, and the absence of a histopathological assessment of microvascular remodeling, including capillary density and vascular changes. The impact of mitral valve abnormalities, particularly SAM, on CMD was not investigated. Additionally, the degree of LGE, as well as the presence of ventricular aneurysms and their relationships with CMD, remains unexplored. Future studies with larger sample sizes and prospective multicenter collaborations are needed to validate and expand upon these findings.

## Conclusion

In patients with oHCM and significant LVOT-PG, CMD was identified through guidewire-based assessment of the LAD. Conducting PTSMA led to an immediate improvement in CMD in most patients. However, some patients exhibited persistent CMD post-PTSMA, which highlights the need for long-term monitoring and further studies to evaluate sustained microvascular function and potential adjunctive therapies.

## Data Availability

The data underlying this article will be shared on reasonable request to the corresponding author.
